# Individual Differences in Sleep Quality: Exploring the Impact of Temperament on Sleep Through Anxiety and Depression

**DOI:** 10.1002/pmh.70100

**Published:** 2026-08-28

**Authors:** Stefano Lasaponara, Angelica Scuderi, Elisa Pellegrini, Ludovica Annarumma, Valentina Piga, Sara Lo Presti, Maurizio Gorgoni

**Affiliations:** ^1^ Department of Psychology “Sapienza” University of Rome Rome Italy; ^2^ Neuropsychology of Attention Lab IRCCS Fondazione Santa Lucia Rome Italy; ^3^ PhD Programme in Behavioural Neuroscience “Sapienza” University of Rome; ^4^ Body and Action Lab IRCCS Fondazione Santa Lucia Rome Italy

**Keywords:** anxiety, arousal, depression, insomnia, personality, sleep, sleepiness, temperament

## Abstract

According to the functional ensemble of temperament (FET) model, individual differences in temperament arise from the balance of neurotransmitter systems also crucial for regulating circadian rhythms and sleep, suggesting a strong neurobiological link between temperament and sleep behavior. The present cross‐sectional study examined whether FET‐defined temperamental traits predict interindividual variation in sleep measures. A total of 394 healthy adults completed the Structure of Temperament Questionnaire (STQ‐77) and validated measures of sleep quality, insomnia severity, daytime sleepiness, and presleep arousal, together with indices of anxiety and depression. Several temperamental traits showed robust associations with sleep outcomes. Neuroticism and impulsivity were associated with poorer sleep quality, greater insomnia severity, and heightened presleep cognitive arousal; these effects were largely mediated by anxiety and depression. Higher social tempo was associated with better sleep and reduced daytime sleepiness. Additional associations emerged for empathy, probabilistic thinking, plasticity, and physical endurance, indicating that orientation, integration, and maintenance FET systems contribute to shaping sleep behavior. These findings indicate that stable, neurochemically grounded temperamental traits may shape interindividual sleep differences, with affective distress as a central mediator. Integrating temperament models into sleep research provides a biologically coherent framework for identifying both risk and resilience factors and informs personalized sleep interventions.

## 1 Introduction

Temperament is defined as constitutionally based individual differences in emotional reactivity and self‐regulation. While early models had no reference to underlying psychophysiological mechanisms, several works have attempted to bridge descriptive taxonomies and physiology through one‐to‐one mappings between neurotransmitters and temperament (Cloninger et al. 1993; Gray 1991; Zuckerman 1994). These frameworks highlighted neurochemical correlates of behavior, but their uni‐ or bidimensional focus could not account for the dynamic interplay among multiple neuromodulators that govern complex affective processes.

The functional ensemble of temperament (FET) model (Trofimova and Robbins 2016) addresses this gap by conceptualizing temperament as emerging from interactions among multiple neurochemical systems. It distinguishes the following three primary neurobiologically driven dimensions: orientation (supporting perceptual and exploratory tendencies), speed of integration (facilitating processing efficiency), and maintenance of behavior (sustaining cognitive and motor activity), reflecting the joint contribution of several neuromodulatory systems (Trofimova 2021; Trofimova and Robbins 2016). Habitual behaviors may be subject to differential regulation when it comes to physical or social‐verbal activities. This model also comprises three emotion‐related traits attributed to opioid regulation.

The FET model has been successfully applied in both clinical and healthy populations (Trofimova and Sulis 2018; Lasaponara et al. 2024; Lozito et al. 2025). By administering the 77‐item Structure of Temperament Questionnaire (STQ‐77) (Rusalov and Trofimova 2007), it is possible to derive individual profiles on 12 biologically based scales.

Literature highlights the role of neurotransmitters associated with temperamental profiles in sleep–wake regulation. Sleep‐ and arousal‐promoting systems represent reciprocal inhibitory circuits based on complex interactions between different neurotransmitters. Sleep initiation is mediated by GABAergic projections from the ventrolateral preoptic nucleus, while wakefulness and cortical activation are sustained by cholinergic, noradrenergic, dopaminergic and serotonergic pathways (Baghdoyan and Lydic 2012). Furthermore, clinical evidence associates an imbalance in sleep–wake neurocircuitry as a factor in the etiology of insomnia (Riemann et al. 2022).

An extensive literature already exists on the association of personality traits based on other established models, like the Big‐5, and several sleep features (e.g., Khazaie et al. 2025). Although informative, this literature is constrained by features inherent to Big Five‐type models, which were derived largely through lexical and factor‐analytic approaches rather than being anchored to specific neurobiological systems. Consequently, the mechanisms linking individual personality dimensions to sleep–wake regulation remain largely underspecified, and broad traits such as neuroticism or extraversion may aggregate heterogeneous facets with potentially distinct physiological substrates (Trofimova and Robbins 2016). Temperament models, and the FET framework in particular, address this limitation by defining traits directly in terms of the neurochemical systems that also regulate arousal and the sleep–wake cycle (Baghdoyan and Lydic 2012), supporting more mechanistically grounded hypotheses than those afforded by descriptive personality taxonomies, and allows broad constructs to be decomposed into specific dimensions (e.g., separating orientation, speed of integration and behavioral maintenance components) that may map more precisely onto distinct sleep‐related outcomes. Although the relationship between temperamental traits and various aspects of the sleep–wake regulation has been widely documented in infancy and childhood (e.g., Ednick et al. 2009; Sorondo and Reeb‐Sutherland 2015), its investigation in adulthood remains limited and based on different theoretical models (Favaretto et al. 2025; Lukowski and Milojevich 2015; Ottoni et al. 2011).

Crucially, this relationship has never been examined within the framework of the FET model.

Here, we hypothesized that individual differences in FET‐derived temperamental traits would predict variability in sleep quality and sleep‐related outcomes. Because FET traits reflect the interplay of arousal‐related neurotransmitter systems, they were expected to influence key aspects of sleep–wake regulation, particularly those associated with arousal modulation. Accordingly, we conducted an online survey in a large Italian sample, administering the STQ‐77 alongside validated measures of sleep quality, insomnia symptoms, daytime sleepiness, and emotional distress (depression and anxiety) as potential mediators.

## 2 Materials and Methods

### 2.1 Participants

The number of participants was determined in advance before data collection through a series of a priori power analyses using G*Power software (Faul et al. 2007).

For the hierarchical multiple linear regression analyses, assuming an effect size of *f* ^2^ = 0.15, a statistical power of 0.80, and a conventional alpha level of 0.05, the required sample sizes were estimated at 146 participants for detecting an *R* ^2^ deviation from zero and 127 participants for detecting significant increases in *R* ^2^. Similarly, in the case of one‐way between‐subjects ANOVAs, a minimum of 53 participants per group (total *N* = 159) was required assuming an effect size of *f*(*U*) = 0.25, a power of 0.80, and an alpha level of 0.05.

A Monte Carlo power analysis simulation was performed to determine the required sample size for mediation analyses involving three parallel mediators, with indirect effects tested using bootstrapped confidence intervals. This analysis, conducted using the tool developed by Schoemann et al. (2017) https://schoemanna.shinyapps.io/mc_power_med/, indicated that a minimum of 200 participants was necessary to achieve a target power of 0.80 with a medium standardized coefficient effect size of 0.30.

Based on these a priori analyses, a sample of 603 volunteers (aged 18–86 years) was recruited through snowball sampling procedure using digital advertising on social media and flyers with a QR code distributed on local bulletin boards in the Rome metropolitan area. All participants were asked to report demographic information consisting in gender and age and reported no history of neurological or psychiatric conditions. Following data collection, 203 participants were excluded due to invalid STQ‐77 responses (Section 2.2.1). Six participants were identified as multivariate outliers according to Mahalanobis distance criteria (Section 2.3) and were also excluded. The final sample comprised 394 participants (100 males, 294 females; mean age: 35 years, SD: 13 years). All provided written informed consent prior to participate in the study, which adhered to the principles of the Declaration of Helsinki and were approved by the local ethical committee.

### 2.2 Experimental Procedure and Tasks

In the present cross‐sectional study, all data were anonymously collected online in an Italian sample from 03/2023 to 06/2024. A digital version of all questionnaires was presented via Google Modules. Each participant received instructions, gave informed consent, declared age ≥ 18 years and provided the explicit agreement to participate in the research through the online form before accessing the survey. They were requested to complete the STQ‐77 questionnaire and sleep‐related questionnaires, followed by questionnaires for the assessment of anxiety and depression. The participant had the possibility to withdraw from the procedure without data saving at any moment. No monetary compensation was provided.

#### 2.2.1 STQ‐77

**FIGURE 1:**
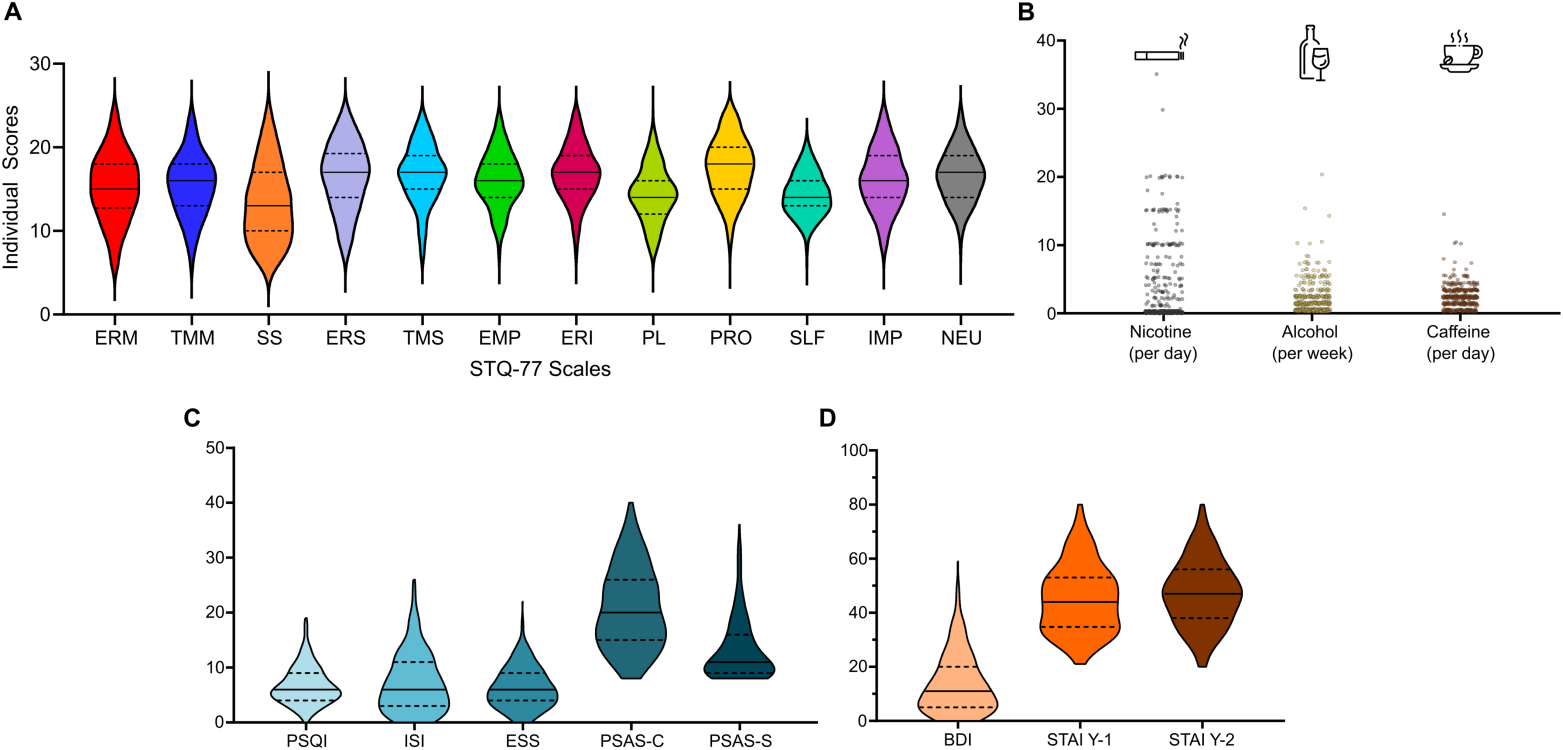
Violin plots representing (**A**) the STQ‐77 scores distribution for each temperamental scale, scores distribution for each sleep‐related dependent variable (**C**) and emotional distress mediators (**D**); continuous black lines indicate median scores; (**B**) individual distribution of psychostimulants usage.

The STQ‐77 (Rusalov and Trofimova 2007) consists of an anamnestic section and 77 items rated on a 4‐point Likert scale (from 1 strongly disagree to 4 strongly agree). The anamnestic section collects information on social status, relatives (nr. of siblings), the use of psychostimulants such as caffeine, alcohol, and nicotine (Figure 1B). The 77 items are grouped into 12 experimental scales (six items each) and one validity scale (five items). Experimental scales are designed to measure 12 biologically based individual traits of behavior (Figure 1A):

1. Physical endurance (ERM): prolonged and sustained physical effort
2. Physical tempo (TMM): speed in physical activity;
3. Sensation seeking (SS): the tendency to engage in risky behaviors;
4. Social endurance (ERS): prolonged and sustained social effort;
5. Social tempo (TMS): speed in speaking, reading or other verbal activities;
6. Empathy (EMP): sensitivity in inferring the emotional state of the other;
7. Intellectual endurance (ERI): prolonged and sustained intellectual effort;
8. Plasticity (PL): the ability to adapt quickly to plan/program changes;
9. Probabilistic thinking (PRO): correct estimation and expectations of the probability of events;
10. Self‐confidence (SLF): the tendency to optimism and confidence in one's abilities;
11. Impulsivity (IMP): lability in emotional reactions, poor immediate control of impulsive behaviors;
12. Neuroticism (NEU): poor tolerance to uncertainty with the expectation of negative situations

The validity scale assesses social desirability tendency. Scores range from 5 to 20, with a score greater than 14 considered invalid.

##### 2.2.1.1 Sleep‐related measures

- The Pittsburgh Sleep Quality Index (PSQI (Curcio et al. 2013)) is a self‐report questionnaire designed to assess sleep quality over the last month. It consists of 19 items and provides a global score of sleep quality, as well as partial scores in seven subscales (subjective sleep quality, sleep latency, sleep duration, habitual sleep efficiency, sleep disturbances, use of sleep medications, and daytime dysfunction). A global PSQI score > 5 indicates poor sleep quality.
- The Insomnia Severity Index (ISI (Castronovo et al. 2016)) is a self‐report questionnaire assessing both nocturnal and daytime insomnia symptoms. It includes seven items (rated on a 0–4 scale). Total scores are interpreted as follows: < 8 indicates absence of insomnia, 8–14 subthreshold clinical insomnia, 15–21 moderate insomnia, and > 21 severe insomnia.
- The Epworth Sleepiness Scale (ESS (Vignatelli et al. 2003)) is a self‐report measure of subjective perceived sleepiness. Participants rate, on a 4‐point scale (0–3), their habitual likelihood of dozing off or falling asleep across eight everyday situations/activities. The score ranges from 0 (no sleepiness) to 24 (severe sleepiness). The Pre‐Sleep Arousal Scale (PSAS (Nicassio et al. 1985)) is a 16‐item self‐reported questionnaire assessing somatic (eight items) and cognitive (eight items) arousal subjectively experienced at bedtime while attempting to fall asleep. Higher scores indicate greater presleep arousal.

##### 2.2.1.2 Emotional distress questionnaires

- The Beck Depression Inventory‐II (BDI‐II (Beck et al. 1996))—is a self‐report questionnaire assessing depressive symptoms. It consists of 21 items rated on a 4‐point scale (0–3), with higher scores indicating greater severity of depressive symptoms. A total score > 13 suggests the presence of clinically relevant depressive disorder
- The State–Trait Anxiety Inventory (STAI‐Y, I–II; Spielberger 1989) is a self‐report questionnaire consisting of 40 items on a 4‐point Likert scale: 20 items assess state anxiety (STAI‐Y I version) and 20 items assess trait anxiety (STAI‐Y II version). Scores ≥ 40 on either scale indicate clinically significant anxiety levels.

#### 2.2.2 Statistical Analyses

To investigate the relationship between temperamental traits and sleep‐related phenomena, we adopted a multistep analytic strategy designed to identify both linear and nonlinear associations and explore potential mediating mechanisms through emotional distress.

In the first step, we examined the presence of linear relationships between temperament and sleep‐related measures, testing whether individual differences in temperamental traits predicted scores on the PSQI, ISI, ESS, and PSAS (Cognitive and Somatic subscales). To this end, six three‐step hierarchical linear regression analyses were performed, with *p* values adjusted applying the Benjamini–Hochberg false discovery rate (FDR) correction. The independent variables were entered in the following order to progressively control for potential confounders:

- Block 1: Demographic variables (age and sex)
- Block 2: Psychostimulants use (daily cigarette and coffee consumption and weekly alcohol intake)
- Block 3: The twelve STQ‐77 temperamental scales

Prior to the regression analyses, multivariate outliers were identified using Mahalanobis distance. Six participants exceeded the critical threshold (*p* < 0.001), as recommended by Tabachnick and Fidell (2007), and were excluded. Collinearity diagnostics (VIF < 2.5 for all predictors) confirmed the absence of multicollinearity. Hierarchical regressions were performed in SPSS (v27).

To complement regression analyses, we tested for potential nonlinear effects of temperament on sleep outcomes. This was based on the theoretical possibility that the relationship between temperament and sleep might not be strictly linear, and that extreme temperamental profiles, rather than gradual variations, might be associated with more pronounced differences in sleep patterns. To this end, participants were categorized into three groups (high, medium, and low) according to STQ‐77 scale scores and established validation cutoffs (scores > 18 = high; < 14 = low) (Rusalov and Trofimova 2007). One‐way ANOVAs were then performed, with group (high, medium, and low trait level) as the between‐subjects factor for each dependent variable. FDR correction was applied to all *p* values, and significant main effects were further examined through Bonferroni‐adjusted post hoc comparisons.

**FIGURE 2:**
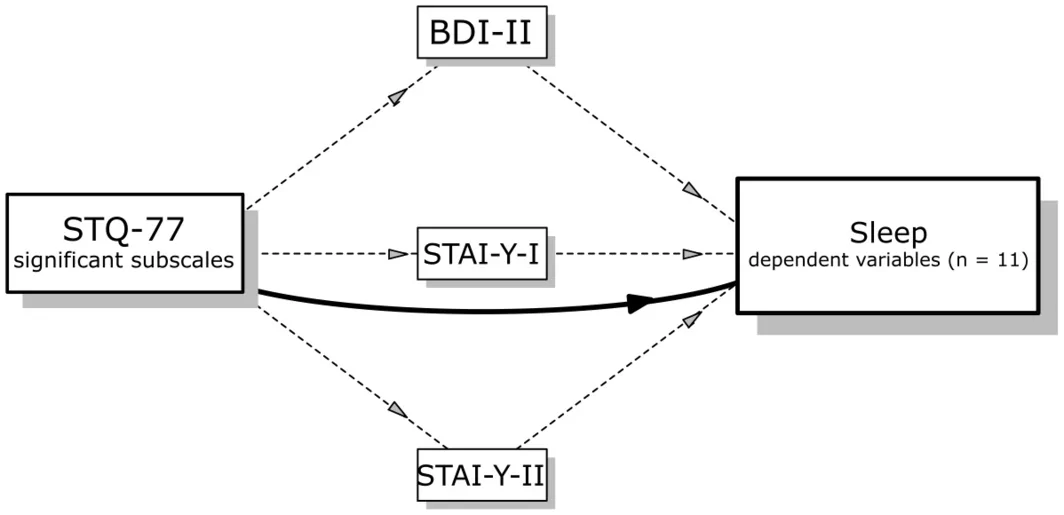
Schematic representation of the hypothesized mediation model with direct (solid bold) and indirect (dashed lines) pathways.

In the third step, we examined whether the associations between temperament traits and sleep‐related outcomes were statistically consistent with indirect pathways through emotional distress, indexed by depressive and anxiety symptoms. Given the cross‐sectional design, these analyses were intended to test theoretically informed patterns of association rather than to establish causal mediation. To this end, parallel mediation analyses were conducted, including only those STQ‐77 scales that showed significant linear and/or nonlinear associations in the previous steps. For each sleep‐related outcome, the selected STQ traits were entered as independent variables, while BDI‐II, STAI‐Y1, and STAI‐Y2 scores were included as mediators (see Figure 2). Direct and indirect effects were estimated using bias‐corrected standardized regression coefficients (*β*), with 5000 bootstrap samples, following the procedure outlined by Biesanz et al. (2010). All mediation analyses were conducted using the jAMM module in Jamovi (v2.3). Missing data were handled excluding participants from specific analyses where the relevant data was unavailable.

### 2.3 Results

#### 2.3.1 PSQI

**FIGURE 3:**
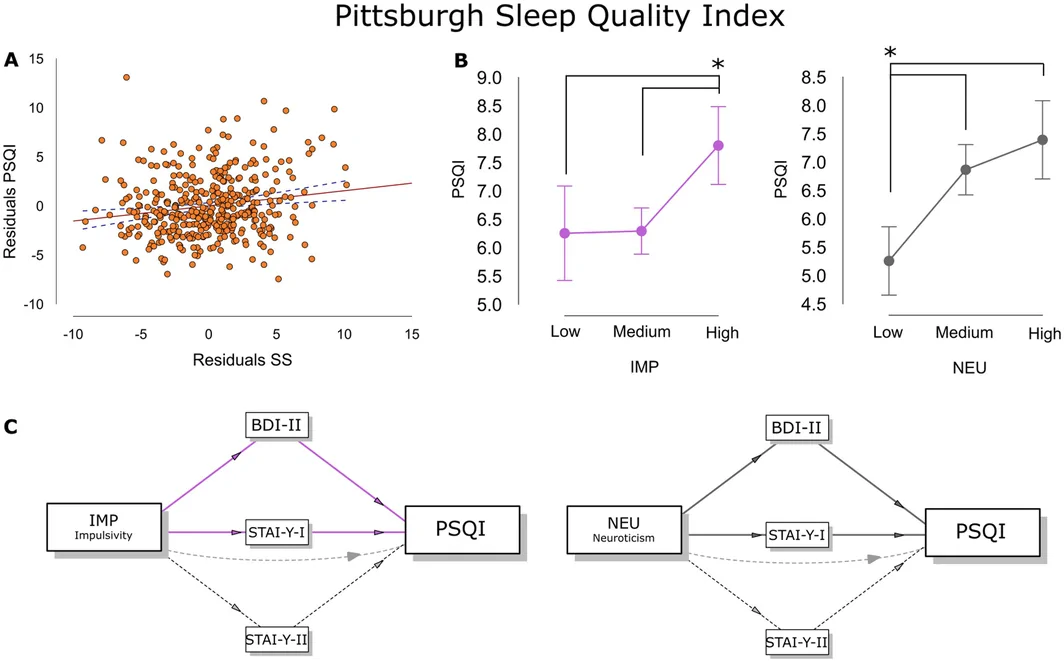
(**A**) Partial correlations between sensation seeking (SS) and Pittsburgh Sleep Quality Index (PSQI) after controlling for covariates. Each dot represents an individual participant, with regression lines indicating the association. (**B**) Mean PSQI scores (± SEM) across low, medium, and high levels of impulsivity (IMP, left panel) and neuroticism (NEU, right panel); *p < 0.05. (**C**) Mediation models testing the indirect effects of impulsivity (left) and neuroticism (right) on PSQI through depressive symptoms (BDI‐II) and state anxiety (STAI‐Y‐I), with trait anxiety (STAI‐Y‐II) included as a covariate. Significant mediation paths are highlighted by solid lines.

Demographic variables (*F*_(2,386)_ = 1.08, *p* = 0.34) and psychostimulants usage (*F*_(5,386)_ = 0.73, *p* = 0.59) did not significantly account for variance in PSQI scores. The inclusion of temperamental traits in the model yielded a significant increase in the portion of explained variance (Δ*R* ^2^ = 0.13, *p* < 0.001). The best‐fitting model accounted for 14.4% of the variance (*F*_(17,386)_ = 3.60, *p* < 0.001) and identified sensation/risk seeking (SS) as the only significant positive predictor (*β* = 0.20, *t*_(393)_ = 3.31, *p* = 0.001; see Figure 3A).

Nonlinear effects were also present for neuroticism (nEU; *F*_(2,391)_ = 9, *p* < 0.001, *η* ^2^ = 0.04) and impulsivity (IMP; *F*_(2,391)_ = 8.80, *p* < 0.001, *η* ^2^ = 0.04; Figure 3B). Specifically, individuals with low neuroticism reported better sleep quality (PSQI = 5.2) compared to those with medium (6.8, *p* = 0.002) and high (7.3, *p* < 0.001) neuroticism scores. Similarly, participants with high impulsivity exhibited poorer sleep quality (PSQI = 7.8) compared to those with medium (6.2, *p* < 0.001) and low (6.2, *p* = 0.010) impulsivity scores. These effects were both positively mediated by depression and anxiety levels (Figure 3C). Specifically, neuroticism was indirectly mediated by BDI‐II (*β* = 0.17, *p* < 0.001) and STAI‐Y‐1 (*β* = 0.08, *p* = 0.020), with no significant direct association with PSQI (*β* = 0.01, *p* = 0.83). Similarly, impulsivity exerted an indirect effect via BDI‐II (*β* = 0.14, *p* < 0.001) and STAI‐Y‐1 (*β* = 0.07, *p* = 0.031), while no significant direct association with PSQI was observed (*β* = 0.07, *p* = 0.12). No significant direct or indirect effects were found for Sensation/Risk Seeking (SS) in relation to PSQI.

#### 2.3.2 ISI

**FIGURE 4:**
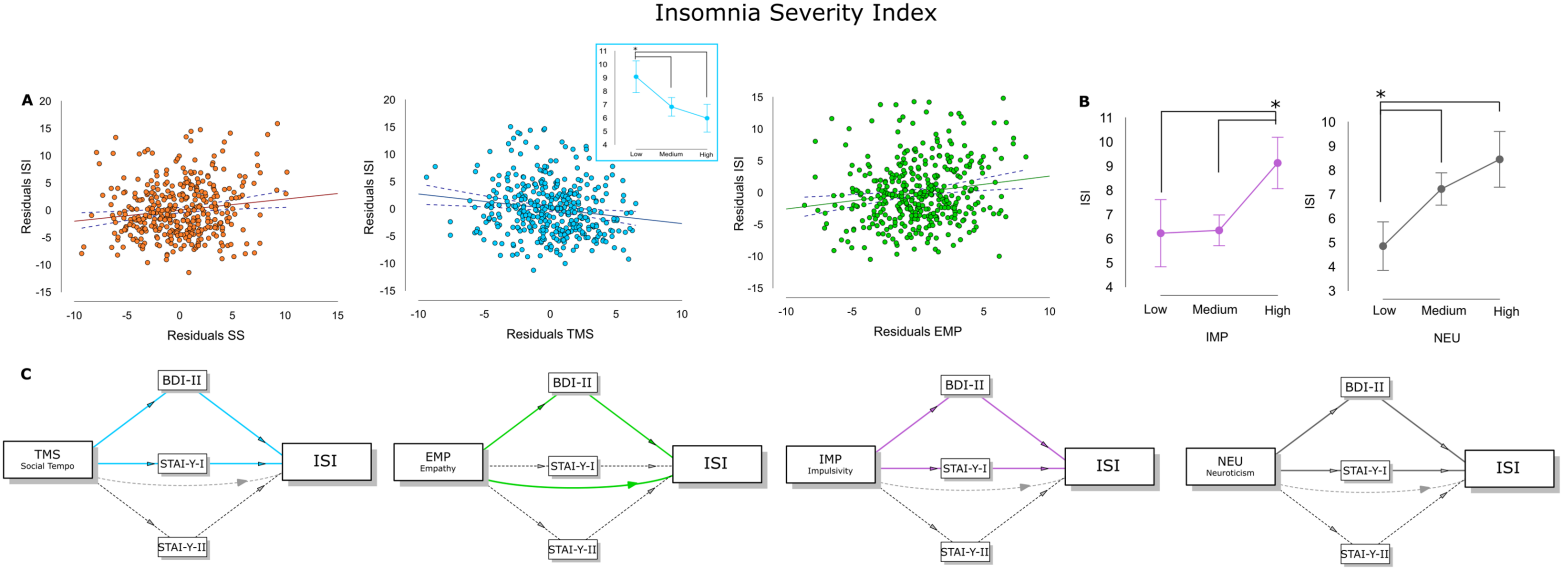
(**A**) From left to right: partial correlations between sensation seeking (SS), Social tempo (TMS), empathy (EMP), and Insomnia Severity Index (ISI) after controlling for covariates. Each dot represents an individual participant, with regression lines indicating the association. (**B**) Mean ISI scores (± SEM) across low, medium, and high levels of impulsivity (IMP, left panel) and neuroticism (NEU, right panel); *p < 0.05. Results for TMS are displayed in the small inset at the top of panel A. (**C**) From left to right: Mediation models testing the indirect effects of TMS, EMP, IMP, and NEU on ISI through depressive symptoms (BDI‐II) and state anxiety (STAI‐Y‐I), with trait anxiety (STAI‐Y‐II) included as a covariate. Significant mediation paths are highlighted by solid lines.

Demographic variables (*F*_(2,386)_ = 0.62, *p* = 0.54) and psychostimulants usage habits (*F*_(5,386)_ = 0.51, *p* = 0.77) did not significantly explain variance in ISI scores. The inclusion of temperamental traits in the model accounted for a significant portion of the variance (Δ*R* ^2^ = 0.17, *p* < 0.001). The final model explained 17.9% of the total variance (*F*_(17,386)_ = 4.74, *p* < 0.001), with sensation seeking (SS; *β* = 0.17, *t*_(393)_ = 2.82, *p* = 0.005) and empathy (EMP; *β* = 0.15, *t*_(393)_ = 3.13, *p* = 0.002) as positive predictors. Social tempo (TMS; *β* = −0.18, *t*_(393)_ = −3.01, *p* = 0.003) emerged instead as a negative predictor (Figure 4A).

Furthermore, individuals with lower propensity for speeded speaking, reading, or other verbal activities (TMS) had higher ISI scores (ISI = 9) compared to those with medium (ISI = 6.8, *p* = 0.002) and high scores (ISI = 5.9, *p* < 0.001; *F*_(2,391)_ = 8.80, *p* < 0.001, *η* ^2^ = 0.04). Consistent with findings for PSQI, participants with low neuroticism had less severe insomnia (ISI = 4.8) compared to those with medium (ISI = 7.2, *p* = 0.004) and high (ISI = 8.4, *p* < 0.001) scores (*F*_(2,391)_ = 9.80, *p* < 0.001, *η* ^2^ = 0.05). Higher impulsivity was also associated with increased insomnia severity (*F*_(2,391)_ = 12.20, *p* < 0.001, *η* ^2^ = 0.06; ISI = 9.1 vs. medium = 6.3, *p* < 0.001; vs. low = 6.2, *p* = 0.001; Figure 4B).

When checking for mediation role of emotional distress (Figure 4C), only Empathy was able to directly (*β* = 0.10, *p* = 0.019) affect ISI scores, although it also exhibited indirect modulation by BDI‐II (*β* = 0.07, *p* = 0.006). In all other cases, the effects of impulsivity, neuroticism and social tempo on ISI were all indirectly mediated by BDI‐II (IMP: *β* = 0.15, *p* < 0.001; NEU: *β* = 0.19, *p* < 0.001; TMS: *β* = 0.13, *p* < 0.001) and STAI‐Y‐1 (IMP: *β* = 0.08, *p* = 0.007; NEU: *β* = 0.10, *p* = 0.004; TMS: *β* = 0.060, *p* = 0.009). No significant direct or indirect effects were found for SS trait (all *p* > 0.090).

#### 2.3.3 ESS

**FIGURE 5:**
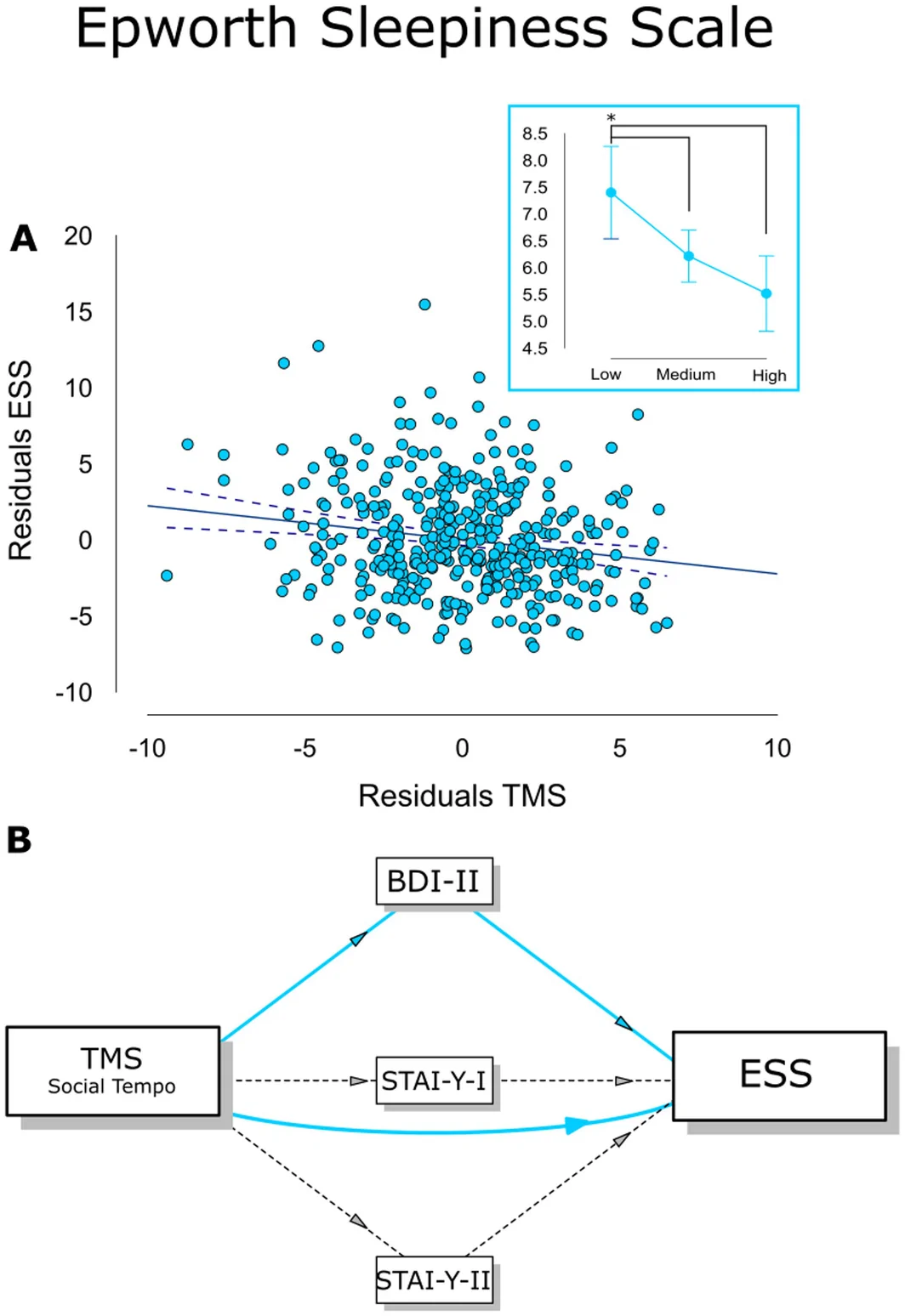
(**A**) Partial correlations between social tempo (TMS) and Epworth Sleepiness Scale (ESS) after controlling for covariates. Each dot represents an individual participant, with regression lines indicating the association. Top right panel, mean ESS scores (± SEM) across low, medium, and high levels of TMS; *p < 0.05. (**B**) Mediation model testing the indirect effects of TMS on ESS through depressive symptoms (BDI‐II) and state anxiety (STAI‐Y‐I), with trait anxiety (STAI‐Y‐II) included as a covariate. Significant mediation paths are highlighted by continuous lines.

Demographic variables (*F*_(2,386)_ = 0.38, *p* = 0.68) and psychostimulants usage habits (*F*_(5,386)_ = 0.57, *p* = 0.72) did not significantly explain variance in ESS scores. The inclusion of temperamental traits in the model accounted for a significant portion of the variance (Δ*R* ^2^ = 0.09, *p* = 0.001). The final model, which explained 9.3% of the total variance (*F*_(17,386)_ = 2.24, *p* = 0.003), identified social tempo (TMS) as the only significant negative predictor (*β* = −0.21, *t*_(393)_ = −3.38, *p* < 0.001; Figure 5A). Moreover, individuals with lower TMS scores exhibited higher daytime sleepiness (ESS = 7.4) compared to those with medium (ESS = 6.2, *p* = 0.030) and high (ESS = 5.5, *p* = 0.002) scores (*F*_(2,391)_ = 6.10, *p* = 0.002, *η* ^2^ = 0.03; Figure 5A). Notably, this effect was both directly (*β* = 0.14, *p* = 0.005) and indirectly mediated by depression levels (*β* = −0.08, *p* = 0.004; Figure 5B).

#### 2.3.4 PSAS‐C

**FIGURE 6:**
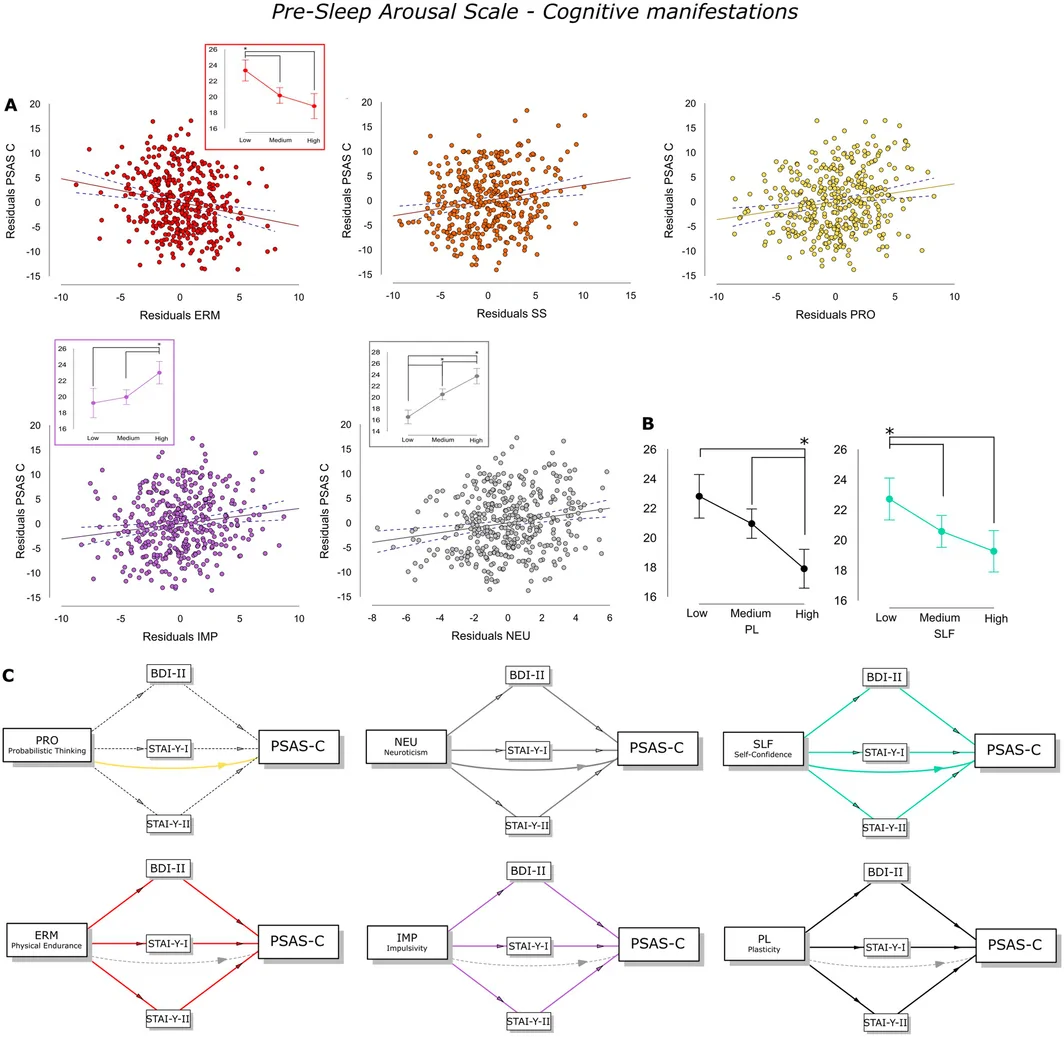
(**A**) From left to right. Top panel: partial correlations between physical endurance (ERM), sensation seeking (SS), probabilistic thinking (PRO) and Pre‐Sleep Arousal Scale–Cognitive (PSAS‐C) after controlling for covariates. Bottom panel: partial correlations between impulsivity (IMP), neuroticism (NEU), and PSAS‐C after controlling for covariates. Each dot represents an individual participant, with regression lines indicating the association. (**B**) Mean PSAS‐C scores (± SEM) across low, medium, and high levels of plasticity (PL, left panel) and self‐confidence (SLF, right panel); *p < 0.05. The results for ERM, IMP, and NEU are displayed in the small inset boxes in panel A. (**C**) From left to right. Top panel: Mediation models testing the indirect effects of PRO, NEU, SLF on PSAS‐C through depressive symptoms (BDI‐II), and state anxiety (STAI‐Y‐I), with trait anxiety (STAI‐Y‐II) included as a covariate. Bottom panel: Mediation models testing the indirect effects of ERM, IMP, PL on PSAS‐C through depressive symptoms (BDI‐II), and state anxiety (STAI‐Y‐I), with trait anxiety (STAI‐Y‐II) included as a covariate. Significant mediation paths are highlighted by solid lines.

Demographic variables (*F*_(2,386)_ = 16.55, *p* < 0.001) significantly explained a portion of variance in PSAS‐C scores, with age emerging as the only significant predictor (*β* = −0.27, *t*_(393)_ = −5.47, *p* < 0.001). Psychostimulants usage habits (*F*_(5,386)_ = 7.21, *p* < 0.001) did not contribute to the explained variance (Δ*R* ^2^ = 0.007, *p* = 0.40). When temperamental traits were added to the model, a significantly larger portion of the variance was accounted for (Δ*R* ^2^ = 0.23, *p* < 0.001). The final model explained 32.0% of the variance (*F*_(17,386)_ = 10.23, *p* < 0.001), and identified SS (*β* = 0.19, *t*_(393)_ = 3.48, *p* < 0.001), PRO (*β* = 0.17, *t*_(393)_ = 3.61, *p* < 0.001), IMP (*β* = 0.15, *t*_(393)_ = 2.94, *p* = 0.003), and NEU (*β* = 0.21, *t*_(393)_ = 3.65, *p* < 0.001) as significant positive predictors, while ERM (*β* = −0.26, *t*_(393)_ = −3.91, *p* < 0.001) was the only negative predictor (Figure 6A).

Moreover, higher presleep arousal was particularly observed in participants with high levels of neuroticism (*F*_(2,391)_ = 23, *p* < 0.001, *η* ^2^ = 0.10; high = 23.7, medium = 20.5, low = 16.5, all *p* < 0.001), impulsivity (*f*_(2,391)_ = 8.60, *p* < 0.001, *η* ^2^ = 0.04; high = 23 vs. medium = 20, *p* < 0.001; vs. low = 19.2, *p* = 0.003), and low physical endurance (ERM: *F*_(2,391)_ = 10.50, *p* < 0.001, *η* ^2^ = 0.05; low = 23.3 vs. medium = 20.2 vs. high = 18.8, all *p* < 0.001). Additionally, individuals with higher plasticity, that is, a greater ability to adapt to change, exhibited lower presleep cognitive arousal compared to those with medium and low scores on this scale (*F*_(2,391)_ = 11.50, *p* < 0.001, *η* ^2^ = 0.06; high = 17.9 vs. medium = 21, *p* = 0.003; vs. low = 22.8, *p* < 0.001; see Figure 8). Conversely, participants with low self‐confidence showed higher presleep cognitive arousal (*F*_(2,391)_ = 6.30, *p* = 0.002, *η* ^2^ = 0.03; low = 22.7 vs. medium = 20.6, *p* = 0.040; vs. high = 19.3, *p* = 0.001; Figure 6B).

Among all these effects probabilistic thinking (PRO: *β* = 0.14, *p* < 0.001) directly modulated PSAS‐C scores (Figure 6C), while neuroticism and self‐confidence influenced presleep arousal both directly (NEU: *β* = 0.09, *p* = 0.035; SLF: *β* = 0.09, *p* = 0.033) and indirectly through BDI‐II (NEU: *β* = 0.09, *p* < 0.001; SLF: *β* = −0.08, *p* = 0.001), STAI‐Y‐1 (NEU: *β* = 0.08, *p* = 0.010; SLF: *β* = −0.08, *p* = 0.006), and STAI‐Y‐2 (NEU: *β* = 0.12, *p* = 0.006; SLF: *β* = −0.14, *p* < 0.001; Figure 6C). Finally, physical endurance, plasticity, and impulsivity (Figure 6C) exerted only an indirect modulation on PSAS‐C scores through BDI‐II (ERM: *β* = −0.06, *p* = 0.004; PL: *β* = −0.06, *p* = 0.003; IMP: *β* = 0.07, *p* = 0.002), STAI‐Y‐1 (ERM: *β* = −0.04, *p* = 0.018; PL: *β* = −0.05, *p* = 0.014; IMP: *β* = 0.07, *p* = 0.011), and STAI‐Y‐2 (ERM: *β* = −0.07, *p* = 0.003; PL: *β* = −0.09, *p* = 0.002; IMP: *β* = −0.11, *p* < 0.001). Also in this case, no significant direct or indirect effects were found for SS trait (all *p* > 0.12).

#### 2.3.5 PSAS‐S

**FIGURE 7:**
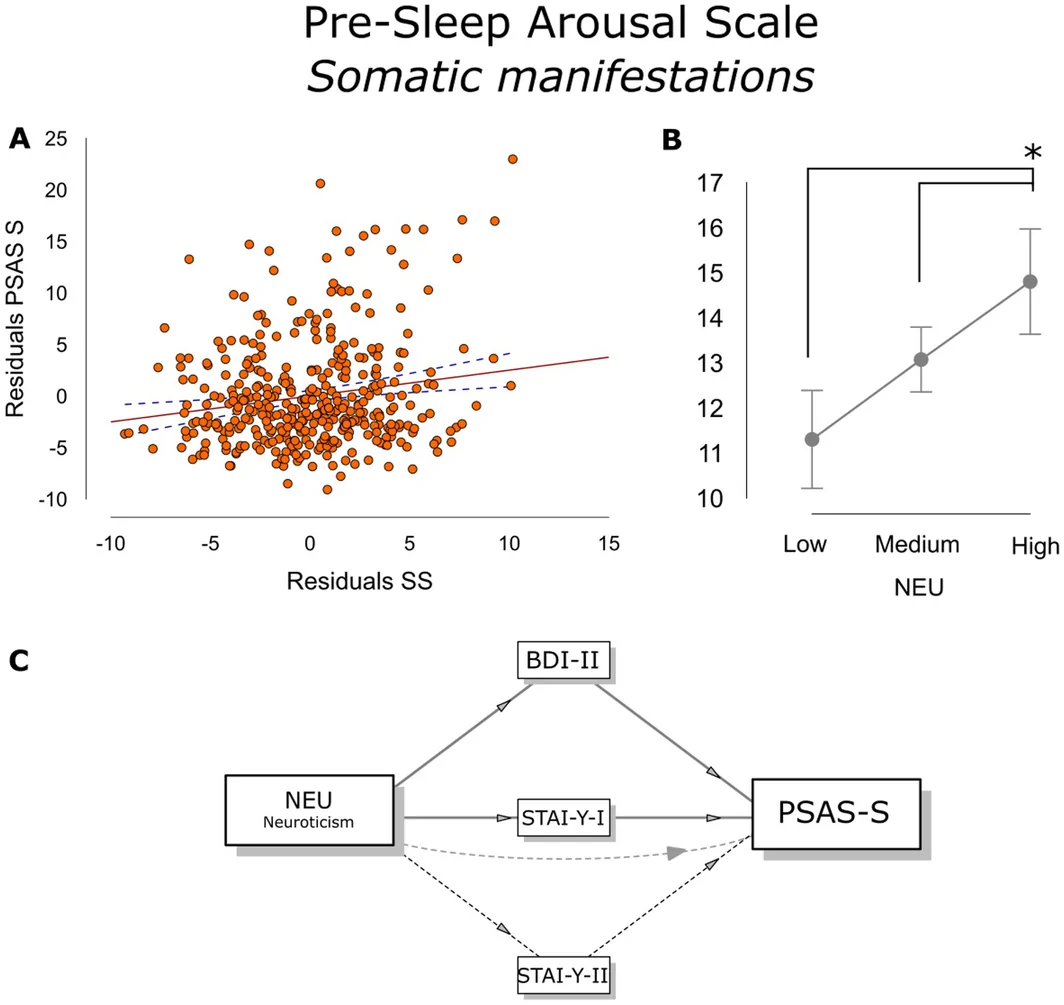
(**A**) Partial correlations between sensation seeking (SS) and Pre‐Sleep Arousal Scale–Somatic (PSAS‐S) after controlling for covariates. Each dot represents an individual participant, with regression lines indicating the association. (**B**) Mean PSAS‐S scores (± SEM) across low, medium, and high levels of neuroticism (NEU); *p < 0.05. (**C**) Mediation model testing the indirect effects of NEU on PSAS‐S through depressive symptoms (BDI‐II) and state anxiety (STAI‐Y‐I), with trait anxiety (STAI‐Y‐II) included as a covariate. Significant mediation paths are highlighted by solid lines.

Demographic variables (*F*_(2,386)_ = 1.82, *p* = 0.16) and psychostimulants usage habits (*F*_(5,386)_ = 1.19, *p* = 0.31) did not explain a significant portion of variance in PSAS‐S scores. Conversely, including temperamental traits substantially improved the model (Δ*R* ^2^ = 0.14, *p* < 0.001), leading to an overall explained variance of 15.3% (*F*_(17,386)_ = 3.91, *p* < 0.001), with SS emerging as the only significant positive predictor (*β* = 0.20; *t*_(393)_ = 3.28; *p* = 0.001; Figure 7A). However, this effect was not significant in the mediation analysis.

At a group level, individuals with higher neuroticism also exhibited higher PSAS‐S scores (*F*_(2,391)_ = 8.70, *p* < 0.001, *η* ^2^ = 0.04: high = 14.8 vs. medium = 13.1, *p* = 0.021; vs. low = 11.3, *p* < 0.001; Figure 7B). This effect was indirectly driven by depression and anxiety, as measured by BDI‐II (*β* = 0.14, *p* < 0.001) and STAI‐Y‐1 (*β* = 0.12, *p* < 0.001; Figure 7C).

### 2.4 Discussion

In the present study, we found that several biologically grounded temperamental traits, as defined by the FET model (Trofimova and Robbins 2016), predicted core aspects of sleep quality, insomnia symptoms, and sleep‐related features, with affective distress frequently emerging as a key mediator. These findings confirm and extend previous evidence by suggesting that neurochemical profiles underlying temperament shape sleep patterns.

#### 2.4.1 Temperament and Sleep Disturbances

##### 2.4.1.1 Behavioural Orientation Traits

Noradrenaline‐mediated behavioral orientation traits are essential for addressing novelty and complexity and exploring behavioral alternatives. Here, physical‐motor, social‐verbal, and mental‐intellectual traits were associated with poorer sleep outcomes, aligning with the established role of noradrenaline in insomnia pathophysiology (Riemann et al. 2012).

Sensation seeking was associated with poorer sleep quality, more severe insomnia symptoms, and presleep arousal. Previous research on this trait and sleep has yielded heterogeneous results across alternative theoretical models (e.g., Akram et al. 2023), while its relationship with presleep arousal has not been examined before. FET‐defined sensation seeking is driven by noradrenergic activity, in interaction with cortisol, dopamine, prolactin, and neuropeptide Y. It represents a behavioral orientation toward intense stimuli or activities, typically accompanied by risk underestimation and linked to heightened hypothalamic–pituitary–adrenal (HPA) axis activation (Trofimova 2021). Noradrenaline, dopamine, and cortisol systems have consistently been implicated in sleep disturbances, hyperarousal, and insomnia (Dressle et al. 2022; Van Someren 2021). Behaviorally, sensation seekers may engage in stimulating activities before bedtime, heightening presleep arousal and increasing vulnerability to sleep difficulties. However, our mediation analyses did not reveal any significant direct or indirect effects for sensation seeking, suggesting that unmeasured factors may better account for its relationship with sleep.

Empathy also predicted higher insomnia severity, with effects partially mediated by depressive symptoms. In the FET model, heightened sensitivity to others' emotions is associated with noradrenergic sensitivity, interacting with opioids and oxytocin. Empathy is a multifaceted construct; its impairment has been associated with problems in social functioning and neuropsychiatric conditions (e.g., Green et al. 2015; Harmsen 2019) but excessive empathy may have negative consequences for psychological well‐being, and empathic distress contributes to psychological problems (Huang et al. 2025). Therefore, a strong empathic trait may increase insomnia risk through its effect on depression and affective dysregulation.

Probabilistic thinking, reflecting the interaction between neocortical noradrenaline, dopamine, serotonin and acetylcholine, represented a novel direct predictor of presleep cognitive arousal. It is defined as the drive to gather information about commonality, frequency, and values of events, to differentiate their features, and to project these in future actions. Its association with increased presleep arousal may reflect the cost of high cognitive vigilance at bedtime, consistent with prior research linking ruminative and worry‐prone tendencies to difficulty disengaging from wakeful thought (Schmidt et al. 2011).

##### 2.4.1.2 Action‐Integration Traits

Dopamine‐mediated action‐integration traits facilitate the coordination of behavioral elements, motivation, and action initiation. We found that plasticity and social tempo were associated with better sleep‐related outcomes.

Social tempo represents the speed and efficiency of prelearned social‐verbal elements of actions. Higher social tempo was associated with reduced insomnia symptoms and daytime sleepiness, with anxiety (for insomnia) and depression (for both insomnia and sleepiness) as mediators. Sleepiness also exhibited a direct association with social tempo. Reduced social tempo has been associated with greater depression and anxiety (Trofimova and Sulis 2018). Social tempo is often discussed within the broader construct of extraversion, which appears negatively correlated with insomnia (Khazaie et al. 2025). However, the concept of extraversion has been criticized as broad and inconsistently defined across personality models (Trofimova and Robbins 2016). Thus, examining specific components such as social tempo may clarify this association. Low social may be mirrored by sluggish cognitive tempo, which includes excessive mind‐wandering, mental‐fogginess, daydreaming, and hypoactivity, behaviors associated with poorer sleep and increased daytime sleepiness (Fredrick et al. 2022) Moreover, several findings suggest a bidirectional relationship between sleepiness and social engagement (Holding et al. 2020). It may be speculated that a stable tendency toward high social tempo, characterized by faster speech processing and efficient management of verbal material, can exert a protective role over excessive sleepiness.

Plasticity, reflecting flexible behavioral adaptation driven by dopamine–serotonin interactions, predicted lower presleep cognitive arousal. Higher plasticity may enhance emotional regulation and reduce perseveration on unresolved thoughts at bedtime. This interpretation aligns with findings linking insomnia to reduced psychological (El Rafihi‐Ferreira et al. 2024) and cognitive flexibility (Ballesio et al. 2019). The relationship between psychological flexibility and sleep quality appears to be mediated by presleep arousal (Mastin‐Purcell et al. 2025). Low flexibility may hinder the downregulation of arousal required for sleep initiation. Our results further suggest that the relationship between plasticity and presleep cognitive arousal is mediated by depression and anxiety, consistent with evidence that reduced cognitive flexibility in major depression (Grant and Chamberlain 2023) and anxiety disorders (Meda et al. 2025) is strongly related to sleep difficulties.

##### 2.4.1.3 Maintenance of Activity Traits

Maintenance of activity traits, primarily supported by serotoninergic and cholinergic systems, sustains the arousal needed for behavioral choices and helps inhibit irrelevant stimuli. In our study, only physical endurance, the ability to sustain prolonged physical activity, negatively predicted presleep cognitive arousal, with effects mediated by depression and anxiety. Low physical endurance has been observed in major depression, consistent with clinically relevant fatigue and reduced activity levels, and comorbid major depression and generalized anxiety disorder (Trofimova and Sulis 2018). Our finding aligns with studies showing that higher physical resilience buffers against stress‐induced sleep disturbances (Kalmbach et al. 2017).

##### 2.4.1.4 Emotional Amplifiers

FET emotional amplifiers are mainly mediated by the opioid receptors. Impulsivity, characterized by delta‐opioid receptor dysregulation, and neuroticism, associated with kappa‐opioid mediated suppression of mu‐opioid receptors, were related to poorer sleep quality, higher insomnia symptoms, and presleep arousal. Conversely, self‐confidence, associated with a neurobiological profile opposite to neuroticism, was linked to reduced presleep cognitive arousal.

Our findings are consistent with extensive literature relating neuroticism to sleep problems (Wang et al. 2025; Akram et al. 2023). In the FET model, neuroticism reflects avoidance of novelty and uncertainty, associated with opioid dysregulation and elevated noradrenergic tone—core mechanisms in insomnia (Riemann et al. 2012). Accordingly, neuroticism predicted poor sleep quality, insomnia severity, and greater presleep arousal, mainly mediated by depression and anxiety, in line with previous findings (Wang et al. 2025). Notably, neuroticism also exerted a direct influence on presleep cognitive arousal, consistent with evidence linking it to dysfunctional sleep‐related beliefs (Zamani et al. 2022). Thus, beyond indirect effects through affective distress, neuroticism may directly predispose to insomnia via cognitive hyperarousal, a key pathophysiological mechanism (Dressle and Riemann 2023).

Impulsivity, defined as the tendency to act under the influence of immediate emotional reactivity, was previously associated with poor sleep quality and insomnia (Akram et al. 2023; Axelsson et al. 2024). Recent evidence further suggests that impulsivity affects sleep quality directly and indirectly via heightened cognitive arousal (Rehman et al. 2025). Within the FET model, impulsivity reflects opioid dysregulation and unstable interactions among dopaminergic systems, BDNF and CREB. As dopaminergic activity promotes wakefulness (Baghdoyan and Lydic 2012), and BDNF alterations are linked to insomnia (Ballesio et al. 2023), impulsivity may reflect a neurochemical imbalance that disrupts sleep architecture. The absence of direct effects in our mediation analyses suggests that the influence of impulsivity on sleep is largely explained by anxiety and depression, supporting the idea that impulsive individuals are prone to affective dysregulation that interferes with sleep.

Finally, greater cognitive arousal was observed in individuals with lower self‐confidence, a trait characterized by a sense of security, dominance, and overconfidence and modulated by opioid–serotonin–dopamine interactions. Low self‐confidence, as measured by the STQ, has been observed in depression, adjustment disorder, schizophrenia, and personality disorders (Trofimova 2021). Unlike neuroticism, which reflects avoidance of uncertainty, self‐confidence may protect against presleep rumination.

#### 2.4.2 Conclusions

This study broadens biologically grounded understanding of individual differences in sleep regulation. Our findings indicate that FET traits predict core aspects of sleep disturbances, with affective distress often serving as an intermediary. Consistently, with the established interaction between temperament and arousal, the strongest effects were observed in relation to presleep cognitive arousal, which was influenced by multiple traits across different functional domains.

These findings open up promising prospects for personalized interventions. Individuals with high impulsivity or neuroticism, whose sleep difficulties are largely determined by affectivity, may benefit most from emotion‐focused cognitive–behavioral strategies. Conversely, those with low endurance or plasticity may be more responsive to chronobiological or physiological interventions targeting circadian alignment and physical recovery. Social tempo and self‐confidence could inform resilience‐focused approaches, guiding preventative strategies in at‐risk populations.

As with all cross‐sectional designs, our study cannot establish causality. Longitudinal and experimental approaches are needed to determine whether temperament traits predispose individuals to specific sleep profiles, or whether sleep patterns themselves contribute to shaping temperament expression. A further limitation concerns the interpretation of the mediation analyses. Although the tested model was theoretically motivated by the assumption that temperament traits represent relatively stable dispositional factors and that emotional distress may act as a more proximal correlate of sleep‐related difficulties, the cross‐sectional design does not allow causal or temporal conclusions. Sleep quality, anxiety, and depressive symptoms are known to be bidirectionally related, and temperament may represent an upstream determinant of all three domains. Therefore, the observed indirect associations should be interpreted as statistically compatible with the proposed model, but not as evidence that emotional distress causally mediates the effect of temperament on sleep. Future longitudinal studies are needed to test the temporal ordering and reciprocal influences among these variables. Reliance on self‐reported measures is another limitation, as these are susceptible to recall and interpretation biases. The online recruitment strategy may introduce an issue of partial self‐selection, limiting the generalizability of the findings. Future studies should integrate objective sleep indices, alongside biomarkers and genetic polymorphisms of monoaminergic systems, to refine the neurobiological mechanisms underlying these associations.

##### Ethical Statement

The final sample comprised 394 participants (100 males, 294 females; mean age: 35 years, SD: 13 years). All provided written informed consent prior to participating in the study, which adhered to the principles of the Declaration of Helsinki and were approved by the Local Ethical Committee (Protocol No. 0002620).

## Disclosure

The authors did not use generative AI technologies for preparation of this work.

## Conflicts of Interest

The authors declare no conflicts of interest.

## Data Availability

The data underlying this article will be shared on reasonable request to the corresponding author.
